# Correlated stochastic epidemic model for the dynamics of SARS-CoV-2 with vaccination

**DOI:** 10.1038/s41598-022-20059-0

**Published:** 2022-09-27

**Authors:** Tahir Khan, Roman Ullah, Basem Al Alwan, Youssef El-Khatib, Gul Zaman

**Affiliations:** 1 Department of Computing, Muscat College, Muscat, Oman; 2 Chemical Engineering Department, College of Engineering, King Khalid University, 61411 Abha, Saudi Arabia; 3 Department of Mathematical Sciences, UAE University, P.O. Box 15551 Al-Ain, United Arab Emirates; 4 Department of Mathematics, University of Malakand, Chakdara, Dir (Lower), Khyber Pakhtunkhawa, Pakistan

**Keywords:** Biological techniques, Mathematics and computing

## Abstract

In this paper, we propose a mathematical model to describe the influence of the SARS-CoV-2 virus with correlated sources of randomness and with vaccination. The total human population is divided into three groups susceptible, infected, and recovered. Each population group of the model is assumed to be subject to various types of randomness. We develop the correlated stochastic model by considering correlated Brownian motions for the population groups. As the environmental reservoir plays a weighty role in the transmission of the SARS-CoV-2 virus, our model encompasses a fourth stochastic differential equation representing the reservoir. Moreover, the vaccination of susceptible is also considered. Once the correlated stochastic model, the existence and uniqueness of a positive solution are discussed to show the problem’s feasibility. The SARS-CoV-2 extinction, as well as persistency, are also examined, and sufficient conditions resulted from our investigation. The theoretical results are supported through numerical/graphical findings.

## Introduction

In Wuhan, China, a respiratory disease outbreak has been started in December 2019. Later, it was identified as a novel coronavirus (COVID-19), known as the SARS-CoV-2 virus. The initial spreading source of the novel disease was an animal. But the pandemic rises from human interaction. Total of 589 million infected individuals have been reported while around 6 and half million deaths occurred till August 13, 2022, around the world. Vaccination is an important weapon against controlling a disease. In the case of the SARS-CoV-2 virus, disease vaccination is very important and there are many vaccines that could be shown their effectiveness. World Health Organization (WHO) investigates that reliable vaccinations program will change the situation. But precautionary measures could be necessary for the time being as it is still doubtful that the vaccine of SARS-CoV-2 provides how many degrees of safeness.

Modeling the real-world problem is an emerging area in the field of science and technology. Mathematical models play a very significant role to explore the dynamics of disease and predicting for future. Also, effective control programs have been forecasted to suggest useful guidelines for health officials. On the basis of these guidelines, it could be easily implemented by taking serious steps to control the disease. Researchers studied epidemiological models to discuss the dynamic behavior of disease by suggesting control mechanism^1–6^. Covid-19 also called the SARS-CoV-2 virus and its vaccination is a challenging task, which attracts the attention of many researchers, (see^7–15^). The reported literature reveals that the mathematical models which have been analyzed are simple and used deterministic approaches. However, the SARS-CoV-2 virus transmission is influenced by different factors (social behavior, age, mobility, virus mutation, etc.,) that can affect the dynamics^16–22^. So from the characteristic of the disease, it could be very interesting if the stochastic approach will be used. A stochastic model has been studied for the novel coronavirus by *Khan et al.,*^23^ very recently, where the random fluctuation is assumed in transmission rate only, while as reported above that due to many factors the SARS-CoV-2 virus is influenced. The main contribution of this paper is to suggest an alternative stochastic model for the SARS-CoV-2 virus, where each population group has its own randomness source, but they are all related by correlation factors. In addition, the correlated suggested model includes the vaccination impact. We formulate a stochastic mathematical model for capturing the realistic nature of the disease. For this, we will extend the work of *Khan et al.,* by incorporating various random sources in which every individual class has various Brownian motions according to the disease characteristics. The vaccination of susceptible individuals is also assumed to investigate the efficiency of vaccination and its role in the minimization of the infection. First, the models will be formulated and then analyzed to discuss the detailed dynamics. We will discuss the existence as well as the uniqueness of the proposed problem to show the well-possed ness and feasibility of the problem. We then show that under what conditions the SARS-CoV-2 virus disease is extinct as well as persists. It is essential to discuss extinction and persistence when investigating virus spread. The aim of this analysis is to determine when the disease will end (extinct) and under which conditions will stay (persist). Finally, all analytical findings will be supported by using some graphical representation in the form of a large-scale numerical simulation by using the Euler-Maruyama scheme. It will be performed via coding the proposed problem with the help of MATLAB and we will show the analytical finding graphically.

## Formulation of the model with fundamental analysis

Let us assume a filtered probability space $(\Omega , {{\mathcal {F}}}_T, ({{\mathcal {F}}}_t)_{t\in [0,T]},P)$ on which lives $W:=(W(t))_{t \in [0,T]}$ with $W(t):=\big (W_i(t):~\text {such that}~i=1,\ldots ,4\big )$, where *W* is a Brownian motion of 4th dimension. Moreover, the natural filtration $({{\mathcal {F}}}_t)_{t\in [0,T]})$ is assumed generated by the Brownian motion *W*. For $k=1,2, 3, 4$, we consider the correlated 1-dimensional Brownian motions $(B_k(t))_{t \in [0,T]}$ given by

$$\begin{aligned} B_k(t):=\sum _{i=1}^{4}\lambda _{ki}(t) W_i(t) \ \ \ \ \text{ where }\ \ \ \lambda _{ki} \text{ are } \text{ constant } \text{ in } [-1,1]. \end{aligned}$$

**Figure 1:**
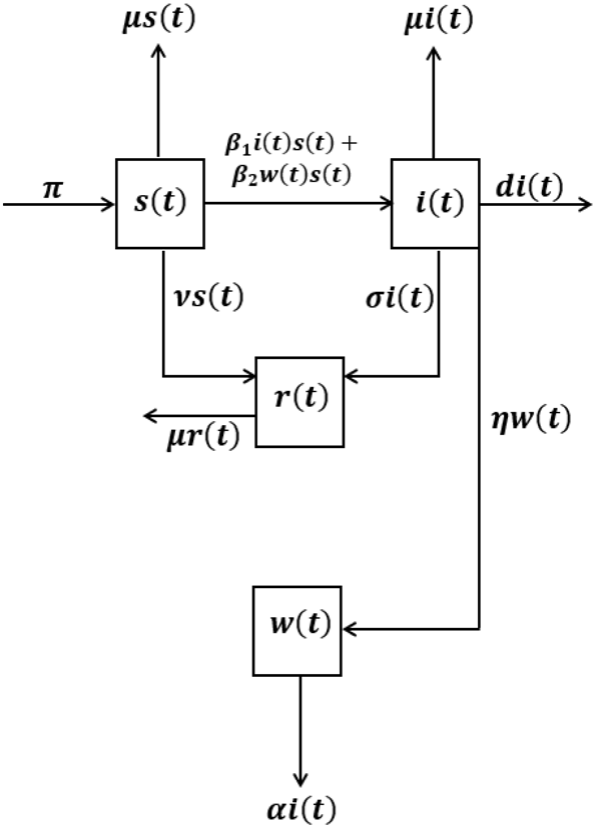
The graph represent the schematic diagram of the proposed model.

We classify the total human population into three human population groups and one class of reservoir. The three population groups are susceptible, SARS-CoV-2 virus infected and recovered, which are symbolized by *s*(*t*), *i*(*t*) and *r*(*t*) respectively, while the reservoir class is denoted by *w*(*t*). The quantity *w* is the environmental reservoir which is an important element in the study of our epidemic model. It represents the concentration of the coronavirus in the environmental reservoir and it includes rates of the infected individuals contributing the coronavirus to the environmental reservoir and the removal rate of the virus from the environment. All the population groups and the reservoir is distributed by different Brownian motions. The schematic diagram for distribution process of the various population groups is given in Fig. 1. Thus we suggest a correlated stochastic epidemic model by the following system:

1 $$\begin{aligned} \begin{aligned} ds(t)&=\left\{ \Pi -\left( \beta _1i(t)+\beta _2w(t)+\mu +v\right) s(t)\right\} dt+\eta _1s(t)dB_1(t),\\ di(t)&=\left\{ \left( \beta _1i(t)+\beta _2w(t)\right) s(t)-(\sigma +d_1+\mu )i(t)\right\} dt+\eta _2i(t)dB_2(t),\\ dr(t)&=\left\{ \sigma i(t)+vs(t)-\mu r(t)\right\} dt+\eta _3 r(t)dB_3(t),\\ dw(t)&=\left\{ \alpha i(t)-\eta w(t)\right\} dt+\eta _4 w(t)dB_4(t). \end{aligned} \end{aligned}$$

The above-proposed model is a generalization of standard epidemic deterministic models. It allows the different quantity of the model to vary stochastically, which mean that the variations are not only time-dependent but also subject to haphazard fluctuations. The random noise detected from real data is considered in the above stochastic model but neglected in deterministic models. In Eq. (1) the various parameters are characterized as: the newborn rate is symbolized with $\Pi$, and $\beta _i, i=1,2$, are routes of disease transmission from the infected human as well as from the reservoir. Moreover, *v* is the vaccination of the susceptible population and $\mu$ is the natural death rate while death from the disease is described with $d_1$. We also symbolize the recovery rate by $\sigma$ and a rate contributed to the virus to the environment by $\alpha$. The removing SARS-CoV virus rate is denoted by $\eta$. If $\lambda _{k1} =1$ for $k=1,2,3,4$, and $\lambda _{ki} =0$ otherwise, then $B_1= B_2=B_3=B_4$ and the model is reduced to the stochastic model studied in *Khan et al.,*^23^. Also, it could be clearly noted that the above system (1) will reduce to the deterministic form, whenever $\eta _1=\eta _2=\eta _3=\eta _4=0$. It can be seen also an extension of^1^. In addition the disease-free and endemic equilibriums of the associated deterministic form of the model are respectively symbolized with $E_0=(s_0,0,0,r_0)$ and $E^{*}=(s^{*},i^{*},r^{*},w^{*})$ with $s_0=\Pi /\mu$, $r_0=v\Pi /dq_1$, where $p_1=\mu +v$. To move towards the endemic equilibrium, we will calculate the *basic reproductive number* first, which is defined to be the average number of secondary infectious produced an infective whenever reached to a totally non-infected population. We assume $X=(i,w)^T$ and $p_2=\sigma +\mu +d_1$, then the deterministic version of the model (1) yields

2 $$\begin{aligned} \frac{dX}{dt}|_{E_0}=-V+F,~\text {and}~F=\left[ \begin{array}{cc} \beta _1s_0 &{} \beta _2s_0 \\ 0 &{} 0 \end{array} \right] ,~V=\left[ \begin{array}{cc} p_2 &{} 0 \\ -\alpha &{} \eta \end{array} \right] . \end{aligned}$$

The *basic reproductive number* is then the spectral radius of $\rho (FV^{-1})$ and consequently looks like

3 $$\begin{aligned} R_0=\frac{\Pi \beta _1}{p_1p_2}+\frac{\Pi \alpha \beta _2}{\eta p_1p_2}. \end{aligned}$$

We use this quantity, to find the components of the endemic equilibrium which may take the form

4 $$\begin{aligned} \begin{aligned}{}&s^{*}=\frac{\eta q_2}{\eta \beta _1+\beta _2\alpha },~i^{*}=\frac{\eta q_1\left( R_0-1\right) }{\beta _1\eta +\beta _2\alpha },~r^{*}=\frac{vs^{*}+\sigma i^{*}}{\mu },~w^{*}= \frac{\alpha }{\eta }i^{*}. \end{aligned} \end{aligned}$$

### Sensitivity analysis

**Figure 2:**
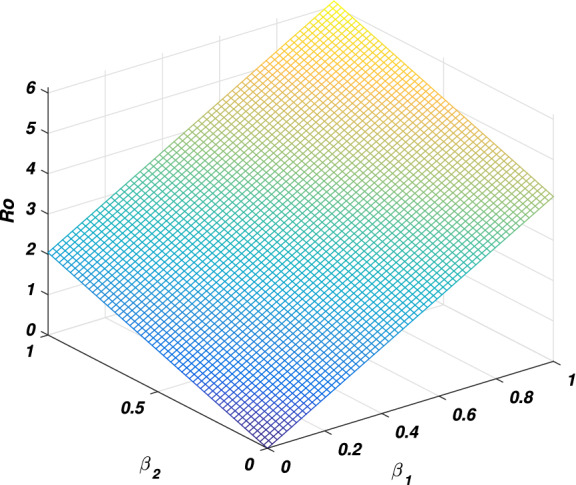
The picture visualizes the variation of the *reproductive number* against $\beta _1$ and $\beta _2$.

**Figure 3:**
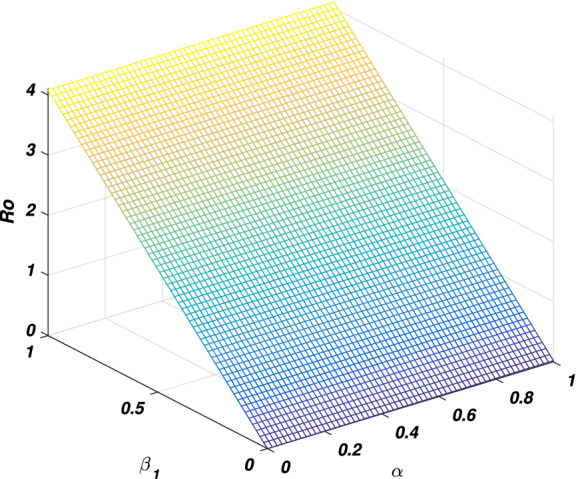
The graph visualizes the variation of the *basic reproductive number* against $\beta _1$ and $\alpha$.

**Figure 4:**
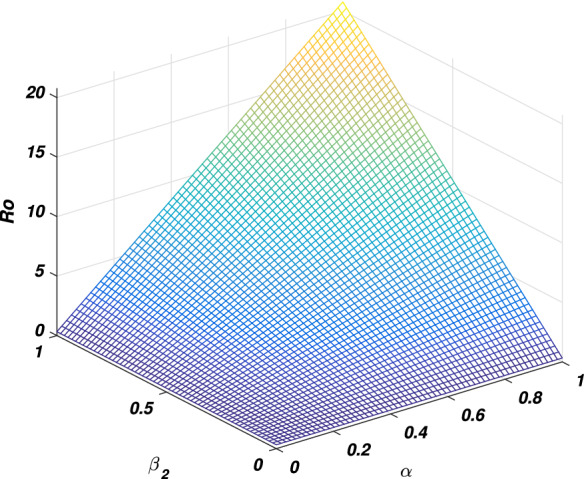
The graph visualizes the variation of the *basic reproductive number* against $\beta _2$ and $\alpha$.

**Figure 5:**
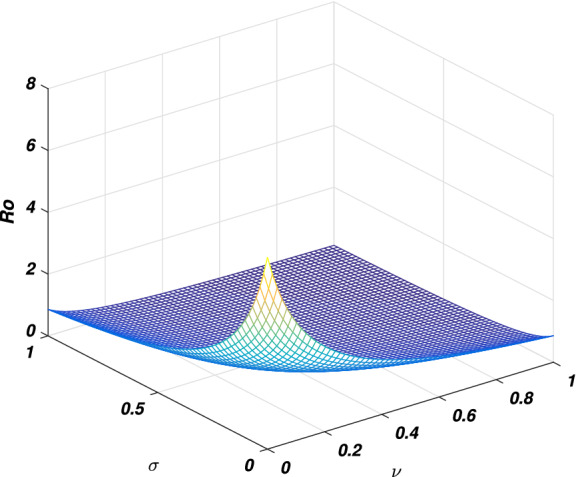
The graph visualizes the variation of the *basic reproductive number* against $\sigma$ and $\nu$.

In every disease the role of the threshold parameter (*basic reproductive number*) is very important and the disease spreads whenever the value of this quantity is more than one and the disease dies out if its value is less than unity. We will discuss the sensitivity of threshold parameter to find the relation between *basic reproductive number* and model parameter. We also calculate the sensitivity indexes that which parameters is how much sensitive to disease control and transmission. Generally the sensitivity index of a parameter say $\phi$ is denoted by $\Upsilon _\phi$ and define as $\frac{\phi }{R_0}\frac{\partial R_0}{\partial \phi }$. By following this formula we calculate the sensitivity indices of model parameters as: $\Upsilon _{\beta _1}=0.9937106918$, $\Upsilon _{\beta _2}=0.006289308180$, $\Upsilon _{\alpha }=0.006289308180$, $\Upsilon _{\nu }=-0.8333333334$ and $\Upsilon _{\sigma }=-0.6862610536$, where the parameters value are taken to be $\Pi =2$, $\beta _1=0.079$, $\beta _2=0.001$, $\mu =0.16$, $\nu =0.8$, $d_1=0.00001$, $\sigma =0.35$, $\alpha =0.1$ and $\eta =0.2$. The biological interpretation of these analyses investigate that the epidemic parameters $\beta _1$, $\beta _2$ and $\alpha$ have a positive influence on the threshold quantity while there is a negative influence with the parameters $\nu$ and $\sigma$. This shows that decreasing in the value of $\beta _1$, $\beta _2$ and $\alpha$, and increasing in the value of $\nu$ and $\sigma$ will decrease the value of the *basic reproductive number*, which is significant in disease elimination. It could be also noted that $\beta _1$ and $\nu$ got the highest sensitivity index and so are the most sensitive parameters to the disease transmission and control. We observed that increasing the value of $\beta _1$ say by 10% would significantly increase the value of $R_0$ by 9.9% as depicted in Fig. 2, while increasing the value of $\nu$ say by 10% decreasing the value of $R_0$ by 8.3% as shown in Fig. 5. Similarly, $\beta _2$ and $\alpha$ collectively effect $R_0$ by 0.1257861636 whenever these parameters are increased or decreased by 10% as depicted by Figs. 3 and 4. The relation between $\sigma$ and $R_0$ is also an inverse as increased $\sigma$ by 10% would decrease the threshold quantity by 6.86% is given in Fig. 5.

### Existence and uniqueness analysis

In this portion of the manuscript the existence of the solution and uniqueness with the positivity of Eq. (1) will be discussed.

It is worth mentioning that the Itô formula is one of the most useful formulas in stochastic calculus. It is utilized, among others, to solve stochastic differential equations. Here, we describe a Multidimensional Itô formula for getting our results by following the book of stochastic calculus^24^.

#### Lemma 2.1

Let $a=(\alpha _1,\ldots ,\alpha _n)$ and $b=(\beta _1,\ldots ,\beta _n)$ represent the adapted processes with square-integrable *n*-dimensional. We consider $X=(X_1,\ldots ,X_n)$, where $X_k$ is driven by the stochastic differential equation and $k \in \{1,\ldots ,n\}$, thus

$$\begin{aligned} dX_k(t)=a_k(t) dt +b_k(t) dB(t) , \ \ \ X_k(0)\in {\mathord {\mathbb R}}. \end{aligned}$$

Let *F* is a given twice continuously differentiable function $f:{\mathord {{\mathbb {R}}}}^n\rightarrow {\mathord {{\mathbb {R}}}}$, then we have

$$\begin{aligned} dF(X(t))=\sum _{k=1}^n\frac{\partial F}{\partial x_k} (X(t))dX_k(t)+\sum _{k,l=1}^n\frac{1}{2}\frac{\partial ^2 F}{\partial x^k x^l}(X(t)) d\left<X_k(t),X_l(t)\right>, \end{aligned}$$

where $$d\left<X_k(t),X_l(t)\right> =b_k(t)b_l(t)dt$$, $$dt=d\left<B(t),B(t)\right>$$, and $$d\left<B(t),t\right> =d\left<t,t\right>=d\left<t,B(t)\right>=0$$.

We use the Lyapunov theory and the virtue of the Itô formula to prove that the solution of Eq. (1) exists globally and is positive. Define

5 $$\begin{aligned} \mathord {{\mathbb {D}}}=\left\{ (s,i,r,w)\in {{\mathord {\mathbb R}}_{+}^4}:s~\text {and}~r>0,~i,w\ge 0,s+i+r+w\le 1\right\} . \end{aligned}$$

The result that discusses the existing analysis of the problem is given by the following theorem.

#### Theorem 2.2

Let (*s*(0), *i*(0), *r*(0), *w*(0)) be the initial classes and assumed to be in ${{\mathord {\mathbb R}}^4_{+}}$, then the solution (*s*(*t*), *i*(*t*), *r*(*t*), *w*(*t*)) of the model (1) is unique as well as remains in ${{\mathord {\mathbb R}}^4_{+}}$ almost surely (*a.s*) i.e.,

$$\begin{aligned} p\left\{ (s,i,r,w)\in \mathord {{\mathbb {D}}}, \forall ~t\ge 0\right\} =1. \end{aligned}$$

#### Proof

We use the procedure as adopted in^25^ and so in the light of this the local Lipschitz continuity property holds for system (1), therefore the solution symbolized by (*s*, *i*, *r*, *w*) of the proposed problem in $[0,\tau _e)$ subject to initial conditions in ${{\mathord {\mathbb R}}_{+}^{4}}$ is unique and local for the explosion time $\tau _e$. Moreover, we investigate that $\tau _e=\infty$ *a.s* as to show the solution globalization. It is assumed that $\kappa _0\ge 0$ is sufficiently large and $\frac{1}{\kappa _0}<N(0)<\kappa _0$, where $N(0)=(s(0),a(0),c(0),r(0))$. We define the stopping time for every $\kappa \ge \kappa _0$ as:

6 $$\begin{aligned} \tau _k=\inf \left\{ t\in [0,\tau _e):\min (s(t),i(t),r(t),w(t))\le \frac{1}{k}~\text {or}~\max (s(t),i(t),r(t),w(t))\right\} . \end{aligned}$$

Further, let $\phi$ is empty set and $\inf \phi =\infty$. Since $\tau _k$ depend on *k* and whenever *k* increasing $\tau _k$ also increasing as *k* increases without bound i.e., tend to $\infty$. Making use of $\lim =\tau _{\infty }$ if $t\rightarrow \infty$ with taking $\tau _{\infty }=\infty$ *a.s* gives that $(s(t),i(t),r(t),w(t))\in {\mathord {\mathbb R}}_{+}^4$, $\forall$ $t\ge 0$ *a.s.* We now only need to show that $\tau _e=\infty$. For this, we use the assumption that for any two constants, $T>0$ and $\varepsilon \in (0,1)$, we have

7 $$\begin{aligned} P\{\tau _{\infty }\le T\}>\varepsilon . \end{aligned}$$

So $k_1\ge k_0$ is an integer that

8 $$\begin{aligned} P\{\tau _{k}\le T\}\ge \varepsilon ,~\text {for every}~ k\ge k_1. \end{aligned}$$

Let *H* is twice continuously differentiable function i.e., $H\in C^2$ and $H:{\mathord {\mathbb R}}_{+}^4\rightarrow {\mathord {\mathbb R}}_{+}$ by

9 $$\begin{aligned} H(s,i,r,w)=s-1-\log (s)+i-1-\log (i)+r-1-\log (r)+w-1-\log (w). \end{aligned}$$

Clearly, $H\ge 0$, so for $0\le T$ and $k_0\le k$, and by the application of the Itô formula leads to the assertion

10 $$\begin{aligned} dH=LHdt+(s-1)\eta _1dB_1+(i-1)\eta _2dB_2+(r-1)\eta _3dB_3+(w-1)\eta _4dB_4. \end{aligned}$$

In Eq. (10), *LH* is defined as

11 $$\begin{aligned} \begin{aligned} LH&=(1-1/s)(\Pi -\beta _1si-\beta _2sw-(\mu +v)s)+\frac{1}{2}\eta _1^2+(1-1/i)(\beta _1si\\&\quad +\beta _2sw-(\mu +d_1+\sigma )i)+\frac{1}{2}\eta _2^2+(1-1/r)(vs+\sigma i-\mu r)+\frac{1}{2}\eta _3^2+(1-1/w)\\&\quad \times (\alpha i-\eta w)+\frac{1}{2}\eta _4^2. \end{aligned} \end{aligned}$$

Simplifying and re-writing the above equation may lead to the following inequality

12 $$\begin{aligned} \begin{aligned}{}&LH\le \Pi +(\beta _1+\alpha )i+\beta _2w+vs+3\mu +v+d_1+\sigma +\eta . \end{aligned} \end{aligned}$$

It could be noted from the fact that $s+i+r+w\le 1$, so the last inequality gives

13 $$\begin{aligned} \begin{aligned}{}&LH\le \Pi +\beta _1+\beta _2+\alpha +2v+3\mu +d_1+\sigma +\eta :=K. \end{aligned} \end{aligned}$$

Plugging Eq. (13) in Eq. (10) we may arrive

$$\begin{aligned} dH\le Kdt+(s-1)\eta _1dB_1+(i-1)\eta _2dB_2+(r-1)\eta _3dB_3+(w-1)\eta _4dB_4. \end{aligned}$$

The integration of both sides reveals that

14 $$\begin{aligned} \begin{aligned} \int _0^{\tau _k\wedge T}dH&\le \int _0^{\tau _kk\wedge T}Kdt+\int _0^{\tau _k\wedge T}(s-1)\eta _1dB_1+\int _0^{\tau _k\wedge T}(i-1)\eta _2dB_2\\&\quad +\int _0^{\tau _k\wedge T}(r-1)\eta _3dB_3+\int _0^{\tau _k\wedge T}(w-1)\eta _4dB_4. \end{aligned} \end{aligned}$$

The expectation of both sides provides

$$\begin{aligned} \begin{aligned} E\bigg [H(s(\tau _k\wedge T),i(\tau _k\wedge T),r(\tau _k\wedge T),w(\tau _k\wedge T))\bigg ]&\le H(s(0),i(0),r(0),w(0))\\&\quad +E\bigg [\int ^{\tau _k\wedge T}_0Kdt\bigg ], \end{aligned} \end{aligned}$$

which implies that

15 $$\begin{aligned} \begin{aligned} E\bigg [H(s(\tau _k\wedge T),i(\tau _k\wedge T),r(\tau _k\wedge T),w(\tau _k\wedge T))\bigg ]&\le H(s(0),i(0),r(0),w(0))+TK. \end{aligned} \end{aligned}$$

Setting a notion of $$\Omega _k={T\ge \tau _k}$$ for all $$k\ge k_1$$. The use of Eq. (7) gives that $$P(\Omega _k)\ge \epsilon$$. Noted that there is at least one $$s(\omega ,\tau _k)$$ or $$i(\omega ,\tau _k)$$ or $$r(\omega ,\tau _k)$$ or $$w(\omega ,\tau _k)$$ equal 1/*k* or *k* for all $$\omega \in \Omega _k$$. Since $$\frac{1}{k}+\log 
k-1$$ or $$-\log k+k-1$$. Hence

16 $$\begin{aligned} \big (s(\tau _k,\omega ),i(\tau _k,\omega ),r(\tau _k,\omega ),w(\tau _k,\omega )\big )\ge \bigg (\frac{1}{k}-1+\log k\bigg )\wedge \big (-\log k-1+k\big ). \end{aligned}$$

So Eqs. (7) and (15) gives

$$\begin{aligned} \begin{aligned}{}&H(N(0))+TK\ge E\bigg [1_{\Omega k(\omega )}H\big (s(\tau _k\wedge T),i(\tau _k\wedge T),r(\tau _k\wedge T),w(\tau _k\wedge T)\big )\bigg ],\\&\quad =E\bigg [1_{\Omega k(\omega )}H\big (s(\tau _k,T),i(\tau _k,T),r(\tau _k,T),w(\tau _k,T)\big )\bigg ]\ge E\bigg [1_{\Omega k(\omega )}\bigg (\log k-1+\frac{1}{k}\bigg )\\&\quad \wedge (-\log k-1+k\big )\bigg ]=\bigg (\log k-1+\frac{1}{k}\bigg )\wedge (-\log k-1+k\big )E\big [1_{\Omega k(\omega )}\big ], \end{aligned} \end{aligned}$$

implies that

$$\begin{aligned} \begin{aligned} H(N(0))+TK&\ge \epsilon \bigg (\log k+\frac{1}{k}-1\bigg )\wedge (-\log k+k-1), \end{aligned} \end{aligned}$$

where $1_{\Omega k(\omega )}$ is a function known indicator function for $\Omega _k(\omega )$. Let $k\rightarrow \infty$ we ultimately obtain $\infty >H\big (N(0)\big )+KT=\infty$, which contradicts, therefore $\infty =\tau _\infty$ *a.s*. $\square$

#### Remark 1

The uniqueness as well as the existence reveals that for any initial compartments $(N(0))\in {{\mathord {\mathbb R}}_{+}^4}$, the unique solution with global axiom $(s,i,r,w)\in {{\mathord {\mathbb R}}_{+}^4}$ almost surly (*a.s*) exists for the proposed problem under consideration as reported by Eq. (1). The previous result can be also proved by the next theorem.

#### Theorem 2.3

Let (*s*, *i*, *r*, *w*) be the solutions of the stochastic differential equations of our model as stated by Eq. (1). The solutions (*s*, *i*, *r*, *w*)

#### Proof

We follow^26^ to discuss the solutions of Eq. (1) which becomes

17 $$\begin{aligned} X_k(t)= & {} \zeta _k(t)\left[ X_k(0)+\int _0^t [\alpha _k(u)-\sum _{j=1}^{m}\theta _{kj}(u)\gamma _{kj}(u)\lambda ^2_{kj}]\zeta _k^{-1}(u)du\right. \nonumber \\&\left. +\sum _{j=1}^{m}\int _0^t\gamma _{kj}(t)\lambda _{kj}\zeta ^{-1}(u)dW_j(u)\right] . \end{aligned}$$

where

18 $$\begin{aligned} \zeta _k(t) = \exp \left[ \int _0^t \left( a_k(u)-\frac{1}{2}\sum _{j=1}^{m}b_{kj}^2(u)\right) du+\sum _{j=1}^{m}\int _0^tb_{kj}(u)dW_j(u)\right] . \end{aligned}$$

Here $k=4$, $m=4$, $\lambda _{kj} =\lambda _{kj}$, $\gamma _{kj}=0$ (for $k,j=1,2,3, 4$) and

$$\begin{aligned} X_1&= s, \ \alpha _1 = \Pi , \ a_1= -\beta _1i(t)-\beta _2w(t)-(\mu +v), \ b_{1j}=\eta _1 \lambda _{1j},\\ X_2 &= i, \ \alpha _2 = \beta _2w(t)s(t), \ a_2= \beta _1s(t)-(\sigma +\mu +d_1), \ b_{2j}=\eta _2 \lambda _{2j},\\ X_3 &= r, \ \alpha _3 = vs(t)+\sigma i(t), \ a_3= -\mu , \ b_{3j}=\eta _3 \lambda _{3j},\\ X_4 &= w,\ \alpha _4 = \alpha i(t), \ a_4=-\eta , \ b_{4j}=\eta _4 \lambda _{4j}. \end{aligned}$$

The Eqs. (17) and (18) show clearly that the solution of our model (1) exists and it is unique and positive if we impose the positivity of the deterministic integral. This ends the proof. $\square$

### Extinction and persistence

In this section, the extinction and persistence analysis of the stochastic model (1) are discussed. We derive the various conditions in the form of some expressions to show permanence and extinction. These expressions containing the model parameters and intensities of noises. Before the formal analysis we define that

19 $$\begin{aligned} \big <g(t)\big >=\frac{1}{t}\int _0^tg(x)dx. \end{aligned}$$

Now it could be described that the persistence of novel coronavirus SARS-CoV-2 is subjected to $\lim \big (\inf \big <i(t)\big >\big )$ and $\lim \big (\inf \big <w(t)\big >\big )$ whenever are positive as *t* increases without bound i.e., to $\infty$. Moreover, the stochastic *reproductive number* of corona dynamical system represented by Eq. (1) is symbolized by $R_0^S$ and define as $R_0^S=R_1^S+R_2^S$, where

20 $$\begin{aligned} R_1^S=\frac{\Pi \beta _1}{p_1\bigg (p_2+\frac{\eta _2^2}{2}\bigg )},~R_2^S=\frac{\Pi \beta _2}{p_1\bigg (p_2+\frac{\eta _2^2}{2}\bigg )}. \end{aligned}$$

Similarly, if

21 $$\begin{aligned} \lim _{t\rightarrow \infty }\inf \int _0^ti(x)dx>0,~{ a.s}., \end{aligned}$$

and

22 $$\begin{aligned} \lim _{t\rightarrow \infty }\inf \int _0^tw(x)dx>0,~{ a.s}, \end{aligned}$$

holds, the epidemic problem represented by Eq. (1) states that the disease will persist. Thus for the extinction analysis of the proposed problem we state the following subsequent result.

#### Theorem 2.4

The SARS-CoV-2 virus will die out exponentially whenever the stochastic *reproductive number* parameter ($R^{S}_0$) is less then unity i.e.,

$$\begin{aligned} \lim _{t\rightarrow \infty } \sup \frac{\log i(t)}{t}\le \bigg (p_1+\frac{1}{2}\xi _{2}^2\bigg )({R_{0}^{S}-1})<0~~\text {{ a.s}}. \end{aligned}$$

Also

23 $$\begin{aligned} \lim _{t\rightarrow \infty }s(t)=\frac{\Pi }{p_1},~\lim _{t\rightarrow \infty }r(t)=\frac{v\Pi }{dp_1},~\lim _{t\rightarrow \infty }w(t)=\lim _{t\rightarrow \infty } i(t)=0,~\text {{ a.s}.} \end{aligned}$$

#### Proof

To prove the result, we integrate the system (1) on both sides which lead to

24 $$\begin{aligned} \begin{aligned}{}&\int _0^tds(x)=\Pi t-\int _0^t\big (\beta _1 i(x)+\beta _2w(x)+p_1\big )s(x)dx+\int _0^t\eta _1 s(x)dB_1(x),\\&\int _0^tdi(x)=\int _0^t\big (\beta _1s(x)+\beta _2w(x)-\sigma -\mu -\mu _1\big )i(x)dx+\int _0^t\eta _2 i(x)dB_2(x),\\&\int _0^tdr(x)=\int _0^t(v s(x)+\sigma i(x)-\mu r(x))dx+\int _0^t\eta _3 r(x)dB_3(x),\\&\int _0^tdw(x)=\int _0^t(\alpha i(x)-\eta w(x))dx+\int _0^t\eta _4 w(x)dB_4(x), \end{aligned} \end{aligned}$$

implies that

25 $$\begin{aligned} \begin{aligned}{}&\frac{s(t)-s(0)}{t}=\Pi -\beta _1 \big<i(t)s(t)\big>-\beta _2\big<w(t)s(t)\big>-p_1\big<s(t)\big>+\frac{\eta _1}{t}\int _0^t s(x)dB_1(x),\\&\frac{i(t)-i(0)}{t}=\beta _1\big<i(t)s(t)\big>+\beta _2\big<w(t)s(t)\big>-p_2\big<i(t)\big>+\frac{\eta _2}{t}\int _0^t i(x)dB_2(x),\\&\frac{r(t)-r(0)}{t}=\sigma \big<i(t)\big>+v \big<s(t)\big>-\mu \big<r(t)\big>+\frac{\eta _3}{t}\int _0^t r(x)dB_3(x),\\&\frac{w(t)-w(0)}{t}=-\eta \big<w(t)\big>+\alpha \big <i(t)\big >+\frac{\eta _4}{t}\int _0^t w(x)dB_4(x). \end{aligned} \end{aligned}$$

The addition of the first two equations of the above system i.e., $\frac{s(t)-s(0)}{t}+\frac{i(t)-i(0)}{t}$ may be written as

26 $$\begin{aligned} \begin{aligned}{}&\frac{s(t)-s(0)}{t}+\frac{i(t)-i(0)}{t}=\Pi -p_1\big<s(t)\big>-p_2\big <i(t)\big >+\frac{\eta _{1}}{t}\int _0^tsdB_{1}(x)\\&\quad +\frac{\eta _{2}}{t}\int _0^ti(x)dB_{2}(x). \end{aligned} \end{aligned}$$

For the sake of simplicity, the notion $\Phi (t)$ will be used in Eq. (26) with some basic algebra we arrive at

27 $$\begin{aligned} \big<s(t)\big>=\frac{\Pi }{p_1}-\frac{p_2}{p_1}\big <i(t)\big >+\Phi (t), \end{aligned}$$

where

$$\begin{aligned} \begin{aligned}{}&\Phi (t)=-\frac{1}{p_1}\bigg [\frac{i(t)-i(0)}{t}+\frac{s(t)-s(0)}{t}\bigg ]+\frac{\eta _1}{t}\int _0^ts(x)dB_{1}(x)+\frac{\eta _{2}}{t}\int _0^ti(x)dB_{2}(x). \end{aligned} \end{aligned}$$

It could be noted from the last result that the limiting value of $\Phi (t)$ is zero whenever *t* approaches $\infty$ i.e.,

28 $$\begin{aligned} \lim _{t\rightarrow \infty } \Phi (t)=0~~\text {{ a.s}}. \end{aligned}$$

The virtue of the Itô formula to the reported epidemic problem (1) gives

29 $$\begin{aligned} \begin{aligned} d\log i(t)&=\beta _{1}s(t)+\beta _{2}\frac{s(t)w(t)}{i(t)}-p_2-\frac{\eta _{2}^2}{2}+\eta _{2}dB_{2}(t). \end{aligned} \end{aligned}$$

The integration of $d\log i(t)$ yields

30 $$\begin{aligned} \begin{aligned} \frac{1}{t} [\log i(t)]_{0}^{t}&=\beta _{1}\big<s(t)\big>+\beta _{2}\bigg <\frac{s(t)w(t)}{i(t)}\bigg >-p_2-\frac{\eta _{2}^2}{2}+\frac{\eta _{2}B_2(t)}{t}. \end{aligned} \end{aligned}$$

It is very much clear from Eq. (5) that $s+i+r+w\le 1$, thus we noted that $\bigg<\frac{s(t)w(t)}{i(t)}\bigg>\le \big<s(t)w(t)\big >\le \big <s(t)\big>$ therefore the above assertion leads to the inequality given by

31 $$\begin{aligned} \begin{aligned} \frac{1}{t} [\log i(t)]_{0}^{t}&\le (\beta _{1}+\beta _{2})\big <s(t)\big >-p_2-\frac{\eta _{2}^2}{2}+\frac{\eta _{2}B_{2}(t)}{t}. \end{aligned} \end{aligned}$$

Using the value of $\big <s(t)\big>$ with some algebraic manipulation and following the well-known *strong law of large number*^27^ i.e., $\lim \sup \frac{\xi _{2}B_{2}}{t}=0~~\text {{ a.s}}$ as $t\rightarrow \infty$ we obtain

32 $$\begin{aligned} \lim _{t\rightarrow \infty } \sup \frac{\log i(t)}{t}\le \bigg (p_2+\frac{\eta _2^2}{2}\bigg )({R_{0}^{S}-1})<0~{ a.s.,} \end{aligned}$$

implies that whenever the condition $R_{0}^{S}<1$ holds, then $\lim i(t)=0$ and so $\lim \big <i(t)\big >=0$ *a.s.,* as $t\rightarrow \infty$. Moreover, the last equation of system (25) implies that

33 $$\begin{aligned} \big<w(t)\big>=\frac{1}{\eta }\left\{ \alpha \big <i(t)\big >+\frac{\eta _4}{t}\int _0^t w(x)dB_4(x)-\frac{w(t)-w(0)}{t}\right\} . \end{aligned}$$

Since the limiting value of *i*(*t*) is zero then $w(t)=0$ whenever $t\rightarrow \infty$, thus the first equation of the system (25) looks like

34 $$\begin{aligned} \big <s(t)\big >=\frac{1}{p_1}\left\{ \Pi +\frac{\eta _1}{t}\int _0^t s(x)dB_1(x)-\frac{s(t)-s(0)}{t}\right\} , \end{aligned}$$

gives that if $t\rightarrow \infty$, $\lim s(t)=\Pi /p_1$. We conclude that the novel disease extinct continuously depends on the value of $R_{0}^{S}$, and ultimately whenever $R_{0}^{S}<1$, it will extinct. $\square$

We have seen from the previous theorem that the virus will die out exponentially if $R_0^S<1$. The next theorem discusses the case when the stochastic reproductive number parameter $R_0^S>1$ is greater than one.

#### Theorem 2.5

If $R_0^S>1$ and $(s_0,i_0,r_0,w_0)$ are any initial population sizes in $\mathord {{\mathbb {D}}}$, then whenever *t* approaches $\infty$, so system (1) holds the conditions given below

35 $$\begin{aligned} i_2\le \lim \inf \big<i(t)\big>\le \sup \big<i(t)\big>\le i_1~\text {and}~w_2\le \lim \inf \big<w(t)\big>\le \sup \big <w(t)\big >\le w_1, \end{aligned}$$

where

36 $$\begin{aligned} \begin{aligned}{}&i_1=\frac{p_1}{p_2(\beta _1+\beta _2)}\left[ \left\{ p_2+\frac{\eta _2^2}{2}\right\} \big (R_0^S-1\big )\right] ,~i_2=\frac{p_1}{\beta _1p_2}\left[ \left\{ p_2+\frac{\eta _2^2}{2}\right\} \big (R_1^S-1\big )\right] ,\\&w_1=\frac{\alpha p_1}{\eta p_2(\beta _1+\beta _2)}\bigg [\left\{ p_2+\frac{\eta _2^2}{2}\right\} \big (R_0^S-1\big )\bigg ],~w_2=\frac{\alpha p_1}{\eta \beta _1p_2}\bigg [\left\{ p_2+\frac{\eta _2^2}{2}\right\} \big (R_0^S-1\big )\bigg ]. \end{aligned} \end{aligned}$$

#### Proof

We noted from Eq. (31) that

$$\begin{aligned} \big <i(t)\big >\le \frac{p_1}{p_2(\beta _1+\beta _2)}\left[ \left\{ p_2+\frac{\eta _2^2}{2}\right\} \big (R_0^S-1\big )\right] +(\beta _1+\beta _2)\Phi (t)+\frac{\eta _2B_2(t)}{t}-\frac{1}{t}[\log i(t)]_0^t. \end{aligned}$$

The application of $\lim$ as *t* approaches $\infty$ with $\sup$ property to the above equation gives

37 $$\begin{aligned} \lim _{t\rightarrow \infty }\sup \big <i(t)\big >\le \frac{p_1}{p_2(\beta _1+\beta _2)}\left[ \left\{ p_2+\frac{\eta _2^2}{2}\right\} \big (R_0^S-1\big )\right] =i_1. \end{aligned}$$

We can also write the following assertion from Eq. (31) that

38 $$\begin{aligned} \begin{aligned} \frac{1}{t} [\log i(t)]_{0}^{t}&\ge \beta _{1}\big <s(t)\big >-p_2-\frac{\eta _{2}^2}{2}+\frac{\eta _{2}B_2(t)}{t}, \end{aligned} \end{aligned}$$

implies

39 $$\begin{aligned} \begin{aligned} \lim _{t\rightarrow \infty }\inf \big <i(t)\big >&\ge \frac{p_1}{\beta _1p_2}\left[ \left\{ p_2+\frac{\eta _2^2}{2}\right\} \big (R_1^S-1\big )\right] =i_2. \end{aligned} \end{aligned}$$

Now the last equation of system (25) can be re-written as

40 $$\begin{aligned} \big<w(t)\big>=\frac{1}{\eta }\bigg [\alpha \big <i(t)\big >+\frac{\eta _4}{t}\int _0^t w(x)dB_4(x)-\frac{w(t)-w(0)}{t}\bigg ]. \end{aligned}$$

Taking $\lim$ as $t\rightarrow \infty$ and $\sup$ of both sides we get

41 $$\begin{aligned} \lim _{t\rightarrow \infty }\sup \big <w(t)\big >\le \frac{\alpha p_1}{\eta p_2(\beta _1+\beta _2)}\bigg [\left\{ p_2+\frac{\eta _2^2}{2}\right\} \big (R_0^S-1\big )\bigg ]=w_1. \end{aligned}$$

On the other hand $\lim$ as $t\rightarrow \infty$ with the application of $\inf$ property Eq. (40) takes the following form

42 $$\begin{aligned} \lim _{t\rightarrow \infty }\inf \big <w(t)\big >\ge \frac{\alpha p_1}{\eta \beta _1p_2}\bigg [\left\{ p_2+\frac{\eta _2^2}{2}\right\} \big (R_1^S-1\big )\bigg ]=w_2. \end{aligned}$$

Thus from Eqs. (37)–(42) it could be noted that $i_2\le \lim \inf \big<i(t)\big>\le \lim \sup \big <i(t)\big >\le i_2$ and $w_2\le \lim \inf \big<w(t)\big>\le \lim \sup \big <w(t)\big >\le w_2$ whenever *t* tend to $\infty$. $\square$

## Numerical simulation

In this section we present the numerical simulation to verify the analytical work. Let us give a short overview to simulate the stochastic differential equations. Let

43 $$\begin{aligned} dX(t)=\alpha (t,X(t))dt+b(t,X(t))dB(t),~~~X(0)=X_0. \end{aligned}$$

Producing a sample *X*(*t*) around *t* with the utilization of the solution of the above equation, we will find *X*(*t*) over a continuous period of time. Making use of the notation ${\tilde{X}}_k$, $B_k$ and ${\tilde{X}}(k\Delta t)$ for simplicity instead of $B(k\Delta t)$. We discretize the Eq. (43) gives

44 $$\begin{aligned} {\tilde{X}}_{\Delta t},{\tilde{X}}_{\Delta t},\ldots ,{\tilde{X}}_{N\Delta t}. \end{aligned}$$

In the above equation, *N* symbolizes the time steps and $\Delta t=T/N$. It could be noted that the application of Itô-Taylor expansion leads to the stochastic Euler Maruyama (SEM) method to simulate the problem under consideration. To retrieve the discretized trajectory of *X*(*t*) from the Eq. (43), we may use the algorithm of Euler Maruyama:

- Simulate $\Delta B_k$ as a normal distributed random variable $N(0, \Delta t)$.
- Putting ${\tilde{X}}_0:=X_0$ and applying ${\tilde{X}}_{k+1}$ by following the formula given below
  45 $$\begin{aligned} {\tilde{X}}_{k+1}=b(k\Delta t,{\tilde{X}})\Delta B_k+\alpha (k\Delta t,{\tilde{X}}_k)\Delta t+{\tilde{X}}_k, \end{aligned}$$
  for $$\Delta B_k=B_{k+1}-B_k$$ and $$k=0,\ldots ,N-1$$.

The stochastic Euler Maruyama technique will be applied for the numerical simulation of the system reported by Eq. (1) which takes the form

46 $$\begin{aligned} \begin{aligned}{}&s_{k+1}-s_k=\left[ \Pi -\beta _1 s_ki_k-\beta _2 s_kw_k-p_1s_k\right] \Delta t+\eta _1s_k\Delta B_{1k},\\&i_{k+1}-i_k=\left[ \beta _1 s_ki_k+\beta _2s_ki_k-p_2i_k\right] \Delta t+\eta _2i_k\Delta B_{2k},\\&r_{k+1}-r_k=\left[ \sigma i_k+\nu s_k-\mu r_k\right] \Delta t+\eta _3r_k\Delta B_{3k},\\&w_{k+1}-w_k=\left[ \alpha i_k-\eta w_k\right] \Delta t+\eta _4r_k\Delta B_{4k}, \end{aligned} \end{aligned}$$

which implies that

47 $$\begin{aligned} \begin{aligned}{}&s_{k+1}=s_k+\left[ \Pi -\beta _1 s_ki_k-\beta _2 s_kw_k-p_1s_k\right] \Delta t+\eta _1s_k\Delta B_{1k},\\&i_{k+1}=i_k+\left[ \beta _1 s_ki_k+\beta _2s_ki_k-p_2i_k\right] \Delta t+\eta _2i_k\Delta B_{2k},\\&r_{k+1}=r_k+\left[ \sigma i_k+\nu s_k-\mu r_k\right] \Delta t+\eta _3r_k\Delta B_{3k},\\&w_{k+1}=w_k+\left[ \alpha i_k-\eta w_k\right] \Delta t+\eta _4r_k\Delta B_{4k}. \end{aligned} \end{aligned}$$

**Figure 6:**
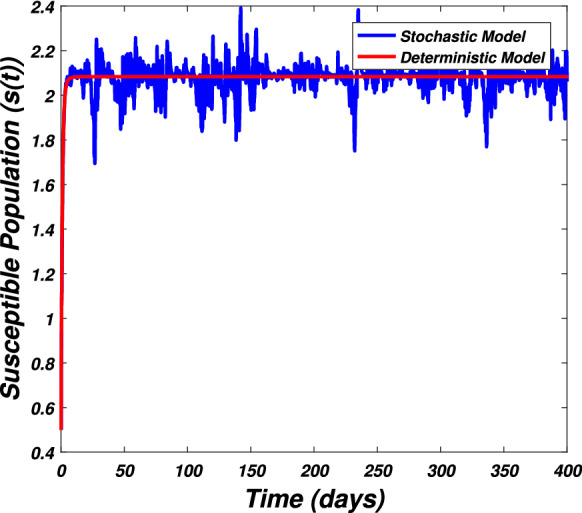
The graph visualizes the temporal dynamics of the epidemic problem described by the model (1) on a large scale for the class of susceptible individuals (*s*(*t*)) in case of extinction. The parameters value used are taken from $S_1$ while (0.5, 0.3, 0.2, 0.1) are assumed to be the initial size of population.

**Figure 7:**
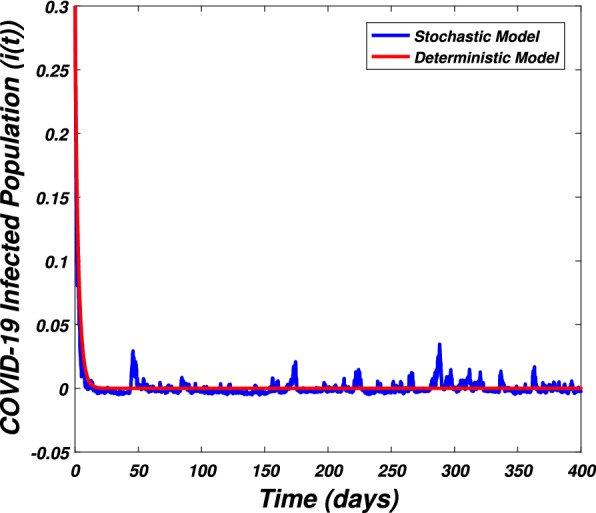
The graph visualizes the time dynamics of the model (1) in case of extinction for the class of infected population (*i*(*t*)) against parametric values taken from $S_1$ and (0.5, 0.3, 0.2, 0.1) are the initial sizes for compartmental population.

**Figure 8:**
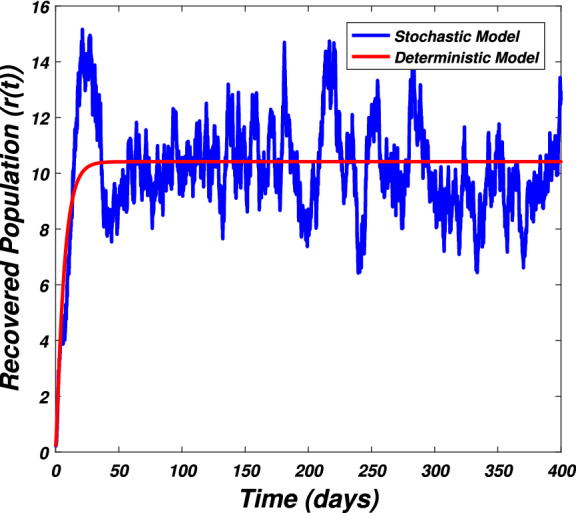
The graph visualizes the temporal dynamics of the model under consideration (1) in the long run for the recovered class (*r*(*t*)) against the parametric value taken from $S_1$ and initial classes (0.5, 0.3, 0.2, 0.1).

**Figure 9:**
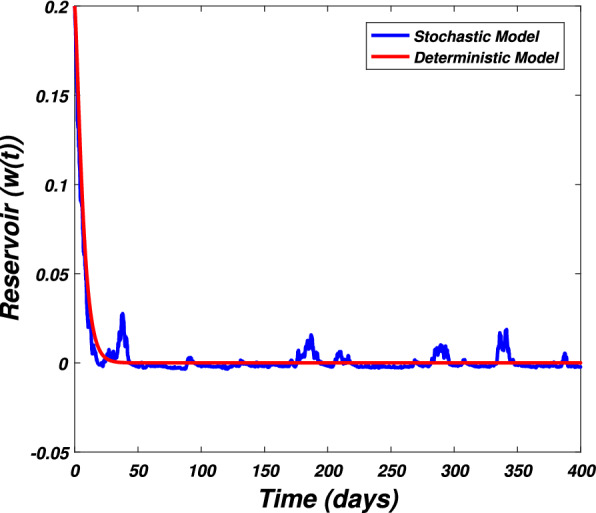
The graph visualizes the time dynamics of the reported model (1) in case of extinction for the reservoir (*w*(*t*)) subject to the parametric values of $S_1$ and (0.5, 0.3, 0.2, 0.1) initial populations.

**Figure 10:**
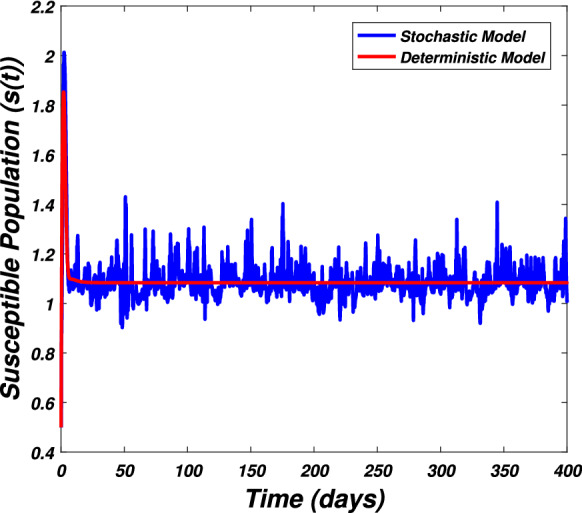
The graph visualizes the dynamics of the epidemic problem described by the model (1) in the case of persistence for the susceptible class (*s*(*t*)) against the values of the parameters taken from $S_2$ and (0.5, 0.3, 0.2, 0.1) are the initial sizes of population.

**Figure 11:**
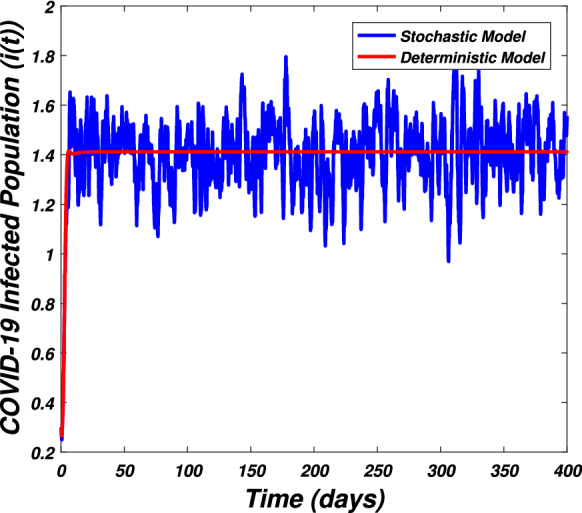
The graph visualizes the persistence of the epidemic problem framed by model (1) for the infected class (*i*(*t*)) against parameters value taken from $S_2$ and various sizes of initial population (0.5, 0.3, 0.2, 0.1).

**Figure 12:**
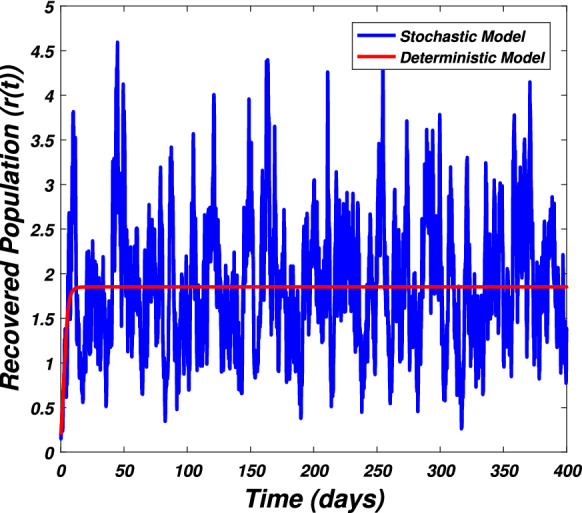
The graph visualizes the time dynamics of the model (1) on large scale for recovered population (*r*(*t*)) against the parametric value of $S_2$ and (0.5, 0.3, 0.2, 0.1) initial population.

**Figure 13:**
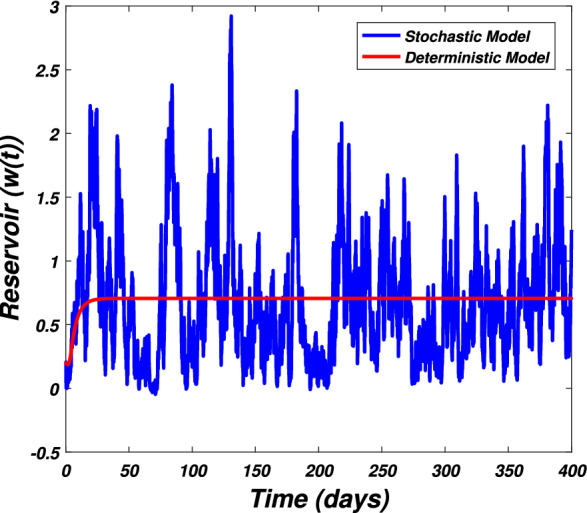
The graph visualizes the large scale numerical simulation of the reservoir class (*w*(*t*)) of the model reported by Eq. (1) against parameters value $S_2$ and (0.5, 0.3, 0.2, 0.1).

Using Matlab software and coding the above algorithm to solve the proposed system. To run our model for large-scale numerical findings we use feasible parameters value with time units of 0 to 400 days. Once we execute the algorithm the following graphs are generated as given by Figs. 6, 7, 8, 9, 10, 10, 11, 12 and 13. This may verify our analytical findings. Moreover, Figs. 6, 7, 8 and 9 demonstrate the temporal dynamics of the susceptible, infected, recovered, and the reservoir respectively, which theoretically investigate that there will be always susceptible and recovered population while the SARS-CoV-2 virus infected population and reservoir will vanishes. This may verify the results of our extinction analysis. Since the disease extinct continuously depends on the *basic reproductive parameter* and whenever $R_0^S<1$ the disease could be easily eliminated. So from the biological point of view, it is very important to keep this quantity low as much as possible to eliminate the disease. On the other hand Figs. 10, 11, 12 and 13 visualize the persistence analysis of the proposed problem. We noted that in this the trajectories of susceptible *s*(*t*), SARS-CoV-2 virus infected (*i*(*t*)), recovered (*r*(*t*)) and reservoir (*w*(*t*)) reveals that the the disease will persist and all these compartments reach to their endemic stage whenever the value of $R_0^S>1$. So special attention is required to make a control mechanism. Since the sensitivity analysis reveals that the disease transmission co-efficient has the highest sensitivity index and a great influence on the threshold parameter therefore minimization of this parameter would significantly decrease the value of the threshold parameter. It could be also noted from the sensitivity index of the vaccination parameter that vaccination has also a strong influence and so increasing the vaccination would strongly decrease the value of *basic reproductive number*. Finally, we also noted a relationship between the noise intensity with disease extinction and persistence i.e., there is a direct relation between the intensity of white noise and extinction while inverse relation between the intensity of white noise and persistence.

## Conclusion

We developed a correlated stochastic epidemic model to discuss the temporal dynamics of the SARS-CoV-2 virus keeping in view the various source of randomness and vaccination of susceptible individuals. We proved the existence and positivity of the solutions which guarantees the well-posedness of the model. In addition, conditions of SARS-CoV-2 extinction analysis and persistence were obtained. A detailed sensitivity analysis has been performed and showed that the disease transmission coefficient and vaccination parameters are the highest sensitive parameters to disease transmission and control. This suggests that the vaccination has a major impact on the dynamics of the SARS-CoV-2. We observed that a rise in this parameter’s value would significantly increase disease extinction. Conversely, the disease persistence reduction is subjected to speedy vaccination, and therefore there is a need for a fast vaccination immunization. Numerical findings were conducted and support the analytical results. Results of this study permit supplementary discussion, such as increasing the impact of the noise. We would encourage researchers to investigate adding jumps to our model.

## Data Availability

All data generated or analyzed during this study are included in this published article.
